# Molecular Dissection of Induced Platinum Resistance through Functional and Gene Expression Analysis in a Cell Culture Model of Bladder Cancer

**DOI:** 10.1371/journal.pone.0146256

**Published:** 2016-01-22

**Authors:** Sisi Wang, Hongyong Zhang, Tiffany M. Scharadin, Maike Zimmermann, Bin Hu, Amy Wang Pan, Ruth Vinall, Tzu-yin Lin, George Cimino, Patrick Chain, Momchilo Vuyisich, Cheryl Gleasner, Kim Mcmurry, Michael Malfatti, Kenneth Turteltaub, Ralph de Vere White, Chong-xian Pan, Paul T. Henderson

**Affiliations:** 1 Department of Internal Medicine, Division of Hematology and Oncology, University of California Davis, Sacramento, California, United States of America; 2 Department of Urology, University of California Davis, Sacramento, California, United States of America; 3 Lawrence Livermore National Laboratory, Livermore, California, United States of America; 4 VA Northern California Health Care System, Mather, California, United States of America; 5 Los Alamos National Laboratory, Los Alamos, New Mexico, United States of America; 6 Accelerated Medical Diagnostics Incorporated, Dublin, California, United States of America; Wayne State University School of Medicine, UNITED STATES

## Abstract

We report herein the development, functional and molecular characterization of an isogenic, paired bladder cancer cell culture model system for studying platinum drug resistance. The 5637 human bladder cancer cell line was cultured over ten months with stepwise increases in oxaliplatin concentration to generate a drug resistant 5637R sub cell line. The MTT assay was used to measure the cytotoxicity of several bladder cancer drugs. Liquid scintillation counting allowed quantification of cellular drug uptake and efflux of radiolabeled oxaliplatin and carboplatin. The impact of intracellular drug inactivation was assessed by chemical modulation of glutathione levels. Oxaliplatin- and carboplatin-DNA adduct formation and repair was measured using accelerator mass spectrometry. Resistance factors including apoptosis, growth factor signaling and others were assessed with RNAseq of both cell lines and included confirmation of selected transcripts by RT-PCR. Oxaliplatin, carboplatin, cisplatin and gemcitabine were significantly less cytotoxic to 5637R cells compared to the 5637 cells. In contrast, doxorubicin, methotrexate and vinblastine had no cell line dependent difference in cytotoxicity. Upon exposure to therapeutically relevant doses of oxaliplatin, 5637R cells had lower drug-DNA adduct levels than 5637 cells. This difference was partially accounted for by pre-DNA damage mechanisms such as drug uptake and intracellular inactivation by glutathione, as well as faster oxaliplatin-DNA adduct repair. In contrast, both cell lines had no significant differences in carboplatin cell uptake, efflux and drug-DNA adduct formation and repair, suggesting distinct resistance mechanisms for these two closely related drugs. The functional studies were augmented by RNAseq analysis, which demonstrated a significant change in expression of 83 transcripts, including 50 known genes and 22 novel transcripts. Most of the transcripts were not previously associated with bladder cancer chemoresistance. This model system and the associated phenotypic and genotypic data has the potential to identify some novel details of resistance mechanisms of clinical importance to bladder cancer.

## Introduction

**Fig 1:**
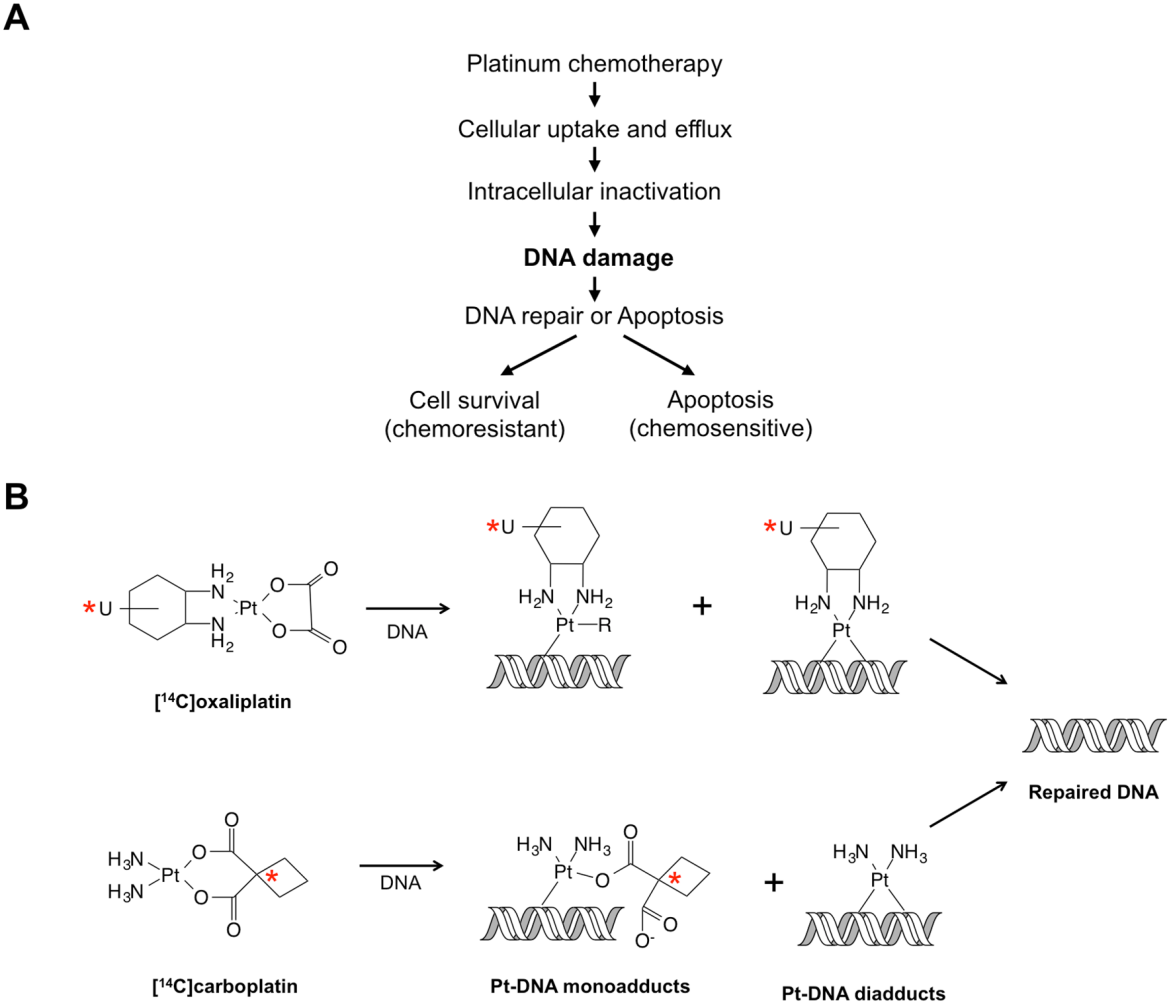
DNA damage as the critical step in Pt-induced cell death. (A) The major pathways of platinum (Pt) drug-induced cell death. After administration, cellular uptake and efflux determines the intracellular accumulation of Pt agents, which can be inactivated by the intracellular thiol-containing molecules. Eventually, Pt agents induce DNA damage, including drug-DNA adducts, which triggers cell cycle arrest and DNA repair. DNA adduct formation and repair determines the fate of cells, although other factors also play important roles, such as pro- and anti-apoptotic proteins. (B) Diagram showing the formation of carboplatin- and oxaliplatin-DNA adducts and the positions of the radiocarbon labels on each drug used for this study in order to enable quantification of drug-DNA adduct formation and repair by accelerator mass spectrometry.

Platinum-based drugs are among the most frequently prescribed anticancer drugs, including cisplatin, carboplatin and oxaliplatin. Cisplatin has been used to treat a broad range of malignancies, such as testicular, lung, ovarian, bladder, head and neck carcinomas, and others. For all platinum-based agents, intrinsic or acquired drug resistance is the major reason for treatment failure (Fig 1A).

The anticancer action of platinum-based drugs is best known for cisplatin, which enters cells by both passive diffusion and active transport. For example, a copper transporter (CTR1) is known to contribute to cisplatin influx and modulates drug sensitivity in vitro [1, 2]. Two copper-efflux-transporting P-type adenosine triphosphates (ATP7A and ATP7B) also mediate intracellular cisplatin levels [3]. Other active transporters include the human organic cation transporter (hOCT) and the human multidrug and toxin extrusion (hMATE), which are found only in certain types of human cells, consistent with the observation that different tissues can vary in their platinum accumulation [4].

Once cisplatin is inside the cell, glutathione (GSH) and other thiols act as reducing agents to quench platinum toxicity. There is high correlation between intracellular GSH levels and resistance to cisplatin *in vitro* [5–7]. Metallothionein proteins are a family of sulfhydryl-rich proteins that participate in heavy metal binding and detoxification and are increased in some cisplatin resistant bladder tumors [8]. Alterations of GSH levels and genes involved in GSH synthesis, as well as metalloproteins, have also been reported for oxaliplatin resistant cancer cell lines [9, 10].

Cisplatin and its aquated or hydroxylated metabolites act as bifunctional alkylating agents for DNA [11]. The resulting drug-DNA adducts block replication and cell division, and activate apoptosis [2]. Other species, such as cisplatin-DNA-protein crosslinks, are also likely to contribute to cisplatin toxicity [12, 13].

Cellular response to carboplatin (see structure in Fig 1B) is thought to be very similar to cisplatin exposure since both drugs form identical crosslink drug-DNA structures, except that carboplatin reacts with DNA more slowly than cisplatin [14]. Clinically, cisplatin and carboplatin have similar, but not identical efficacy, likely owing to differences in biochemistry and dosing regimens.

Oxaliplatin (Fig 1B) acts similarly to cisplatin by exerting its toxicity via drug-DNA adduct formation [15–17]. Since oxaliplatin-DNA adducts have different chemical and biological properties from cisplatin-DNA adducts, it does not show full cross-resistance with cisplatin and is more efficient in, for instance, inhibiting DNA synthesis [18–20]. Also, differences between cisplatin and oxaliplatin have been described for intracellular cascades induced by drug-DNA damage related to apoptosis and cell cycle arrest [21].

Nearly all platinum-based drug-DNA adducts are substrates for nucleotide excision repair (NER) [2]. Increased DNA repair rates have been documented to correlate with resistance to platinum drugs [5, 22–25]. Platinum-DNA adducts are also substrates for the DNA mismatch repair system (MMR). MMR proteins have a much higher affinity for cisplatin- than for oxaliplatin-DNA adducts [26, 27]. It has been reported that a defective MMR activity results in an increased resistance of cell lines to cisplatin, but not to oxaliplatin [19], which may explain the relative efficacy of oxaliplatin in colorectal cancers that are often defective in MMR [28, 29].

Molecular pathway analysis has been highly successful in laboratory research for elucidating platinum-based drug resistance mechanisms. However, the resulting molecular signatures of drug resistance are rarely applicable to the clinic. One major reason for the huge gap between the laboratory research and clinical application is the highly complex nature of resistance mechanisms against cytotoxic drugs. At the cellular level, over 700 genes are involved in cellular response to platinum-based treatment [30].

This complexity motivated us to generate a nearly isogenic bladder cancer cell line for the purpose of extending the mechanistic analysis of platinum resistance to bladder cancer, for which platinum-based treatment is first line in Stage II and higher disease [31, 32]. We present in this paper a phenotypic and genotypic analysis of a parental bladder cancer cell line 5637 and a daughter cell line that was rendered resistant to platinum-based drugs by exposure to increasing concentrations of oxaliplatin over several months. We hypothesized that this pair of cell lines would exhibit differences in platinum drug accumulation, intracellular inactivation and drug-DNA formation and repair consistent with their sensitivity to each drug, and that gene expression analysis of these nearly isogenic cell lines will result in reasonable number of testable hypotheses that may be specific to bladder cancer. The cell lines were tested for sensitivity to several chemotherapy agents commonly used in the treatment of bladder cancer. The cell lines were also assessed in detail with respect to mechanistic differences in response to [^14^C]oxaliplatin and [^14^C]carboplatin. The ^14^C tracer enabled determination of drug uptake and efflux by liquid scintillation counting (LSC) and drug-DNA adduct formation and repair by accelerator mass spectrometry (AMS). The cell lines were also analyzed for RNA transcript expression changes by RNAseq, which led to the identification of several known and some novel transcripts with respect to platinum-based drug resistance. The contribution of the genes represented by these transcripts to chemoresistance is largely unknown. Elucidation of these mechanistic details in subsequent work may ultimately help design personalized therapy to overcome chemoresistance and guide the development of novel therapeutic agents against bladder cancer.

## Materials and Methods

### Drugs

Oxaliplatin (5 mg/ml) was purchased from Sanofi-Aventis (Bridgewater, NJ, USA) and ^14^C-labeled oxaliplatin ([^14^C]oxaliplatin) (specific activity of 58 mCi/mmol) and [^14^C]carboplatin (54 mCi/mmol) were purchased from Moravek Biochemicals. Mixtures of radiocarbon-labeled and non-labeled oxaliplatin or carboplatin (USP Pharmaceutical Grade) were used in order to minimize the usage of radiocarbon, and achieve the different specific activities required for this study. Drug solutions were prepared immediately prior to use. Other drugs were obtained from the UC Davis Cancer Center Pharmacy (USP Pharmaceutical Grade).

### Cell lines

Human bladder cancer cell lines were purchased from the American Type Culture Collection (ATCC, Manassas, VA) and cultured with the recommended medium unless otherwise specified. To develop Pt-resistant sub-cell lines, 5637 (HTB-9) cells were cultured around the IC_50_ concentrations of oxaliplatin intermittently with stepwise increase of oxaliplatin concentration. The oxaliplatin concentrations used ranged from 1.5 μM to 15 μM, which are physiologically relevant considering maximum plasma concentration in humans is approximately 10 μM [33]. After 10 months of culture, the resistant sub-cell line 5637R was developed. To confirm that 5637R originated from the parental 5637 cell line, samples of both cultures were sent to the ATCC Cell Line Authentication Service for cell verification per the ATCC protocol. Specifically, fifteen short tandem repeat (STR) loci plus the gender determining locus, amelogenin, were amplified using the commercially available PowerPlex^®^ 16HS Kit from Promega. The cell line sample was processed using the ABI Prism^®^ 3130 xl Genetic Analyzer. Data were analyzed using GeneMapper ID v 3.2 software (Applied Biosystems). Appropriate positive and negative controls were used throughout the test procedure.

### MTT Assay to determine IC_50_

The IC_50_ values were determined after incubating cells for 72 hours with different concentrations of chemotherapeutic agents commonly used in treating bladder cancer, as previously described [34].

### Oxaliplatin and carboplatin exposure and AMS analysis

Cells were seeded in 60 mm dishes at a density of 1 x 10^6^ cells/dish and allowed to attach overnight in a 37°C humidified atmosphere containing 5% CO_2_. At hour 0, cells were dosed and incubated with 10 μM oxaliplatin supplemented with 5,000 dpm/ml of [^14^C]oxaliplatin or 100 μM carboplatin,supplemented with 50,000 dpm/mL [^14^C]carboplatin. The 24-hour incubation was used to mimic the *in vivo* oxaliplatin half-life (16.8 hours) in patients [35, 36]. The cells were then washed twice with phosphate-buffered solution (PBS) and maintained thereafter with drug-free culture media. DNA was harvested at time points over 24–48 hours, as indicated, and purified with a Promega Wizard DNA purification kit. Ten micrograms of DNA per sample was converted to graphite and measured by AMS for ^14^C quantification as previously described [37]. Triplicate sets of AMS experiments were performed and the data was plotted as time vs oxaliplatin-DNA adducts per 10^8^ nt.

### Determination of intracellular glutathione levels

Intracellular total glutathione (GSH) level was detected with a colorimetric GSH detection kit per manufacture’s protocol (BioVision, Mountain View, CA). Approximately 10^7^ cells were washed with ice-cold PBS, and lysed in GSH lysis buffer. After incubation on ice for 10 minutes, sulfosalicylic acid solution was added, and supernatant was collected for measurement of absorbance at 410 nm. GSH standard included in the kit was used to generate a standard curve for determining the sample GSH concentrations.

### Statistics

We used quantitative summaries of the DNA damage, IC_50_ and AUC (area under curve) values, separately by experiment, cell line and time (mean and standard deviation). Statistics were calculated with n = 3 for each cell line. ANOVA analysis of IC_50_ and AUC data were based on a one-sided *t*-test. All tests were at an experiment-wise error rate of 0.05 and all analyses used SAS/STAT^®^ or MedCalc^®^ software.

### RNAseq and qRT-PCR

Total RNA was isolated using Qiagen RNeasy mini kit. Co-purified genomic DNA was quantified using an 18S gene-specific quantitative PCR assay with human genomic DNA as the quantity standard. Since total RNA had high levels of genomic DNA contamination (predicted to account for 15–68% of total reads), an additional DNase treatment step was added to the RNA purification protocol. This step reduced the DNA contamination to levels expected to produce <0.33% of total reads. rRNA was depleted from the samples using Epicentre's RiboZero H/M/R kit. Sequencing libraries were made from 40 ng of rRNA depleted RNA using Epicentre's ScriptSeq v2 RNA-Seq Library Preparation Kit. These samples were sequenced on 1/3 of a HiSeq PE 101 lane each at Los Alamos National Laboratory.

Raw sequencing data were processed by CASAVA 1.8 software (Illumina; San Diego, CA) and trimmed for quality (Q_30_, Phred scale). Analysis of RNA-Seq data was performed using a standard TopHat-Cufflinks workflow with human genome assembly (Feb. 2009, GRCh37/hg19) [38, 39]. The expression of a transcript was considered significantly regulated if FDR (p-value corrected for multiple testing) was less than 0.05.

For qRT-PCR, RNA was isolated from subconfluent dishes using the Qiagen RNeasy Mini Kit according to the manufacturer’s instructions. cDNA was synthesized using the Thermo Scientific RevertAid RT kit. qRT-PCR was performed using the EconoTaq PLUS 2X master mix on a BioRad CFX96 Real-Time System instrument. The following primers were used: TSPAN7 (ACCAAACCTGTGATAACCTGTCT, AGGGAGATATAGGTGCCCAGA), AKR1C2 (ATTGGAATGACATACTGCATCCT, GTTCAACCGTTTCTTACCTGTGG), AKR1C1 (CGCCTGCAGAGGTTCCTAAAA, ATCAATATGGCGGAAGCCAG), CYR61 (CCCGTTTTGGTAGATTCTGG, GCTGGAATGCAACTTCGG), HTRA1 (TCCCAACAGTTTGCGCCATAA, CCGGCACCTCTCGTTTAGAAA), and AQP3 (CCGTGACCTTTGCCATGTG, CGAAGTGCCAGATTGCATCATAA). Any RNA seq data not presented in the paper is available online at http://www.ncbi.nlm.nih.gov/sra. Raw RNA seq data have been assigned accession ID numbers SRR1820076 and SRR1820077 for the 5637 parental line; and SRR1820079 and SRR1820080 for the 5637R line (representing two independent experiments for each cell line).

## Results

The generation of the 5637R cell line via induced drug resistance is described below, along with a variety of phenotypic and genotypic characterizations. Unless otherwise noted, comparisons between the two cell lines are presented in the order of 5637R versus 5637, respectively.

### Induction of Platinum Drug Resistance

The 5637R cell line was developed over 10 months of culture with a stepwise increase in the concentration of oxaliplatin in the media. The cytotoxicity of oxaliplatin to the 5637R line decreased by approximately 10-fold compared to the parental cell line (IC_50_ of 26.1 μM versus 2.45 μM, p<0.0001, Table 1). Unexpectedly, we were unable to develop a resistant 5637 derivative upon extended exposure to carboplatin. To ensure that 5637R originated from the parental 5637 cells, one aliquot of each cell line was sent to ATCC for determination of clonal fidelity. The 15 short tandem repeat (STR) loci plus amelogenin of the 5637 cell line used for this study were an exact match for the ATCC human cell line 5637 (HTB-9) in the ATCC database. The 5637 line had three alleles that 5637R lacked while all other alleles examined were the same for both cell lines, suggesting that 5637R is a derivative of 5637.

**Table 1:** Comparison of drug IC_50_ values for 5637 and 5637R cells.

Cell lines	Oxaliplatin	Carboplatin	Cisplatin	Gemcitabine	Doxorubicin	Methotrexate	Vinblastine
**5637 (μM)**	2.45	24.34	0.59	0.12	0.27	1.24	0.000605
**5637R (μM)**	27.27	72.18	2.99	1.44	0.29	2.01	0.000595
***P values***	*<0*.*0001*	*<0*.*0001*	*0*.*049*	*0*.*0015*	*0*.*45*	*0*.*18*	*0*.*48*

### Chemotherapy drug cytotoxicity

Cells were cultured with a range of concentrations of cisplatin, carboplatin, gemcitabine, doxorubicin, methotrexate and vinblastine for 72 hours followed by assessment of viability by the MTT assay (Mean values reported in Table 1). These drugs were chosen because of their frequent use in the treatment of bladder cancer. The 5637R cell line was also more resistant to cisplatin, but to a much lesser extent than for oxaliplatin (IC_50_ 2.99 μM versus 0.59 μM for 5637, p = 0.049), and to carboplatin (IC_50_ = 72.18 μM versus 24.34 μM, p<0.0001). It was also more resistant to gemcitabine (IC_50_ = 1.44 μM versus 0.12 μM, p = 0.0015), but both cell lines were equally sensitive to doxorubicin (IC_50_ = 0.27 versus 0.29 μM, p = 0.45), methotrexate (IC_50_ = 1.24 μM versus 2.01 μM, p = 0.18) and vinblastine (IC_50_ = 0.61 nM versus 0.60 nM, p = 0.48).

### Uptake and efflux

**Fig 2:**
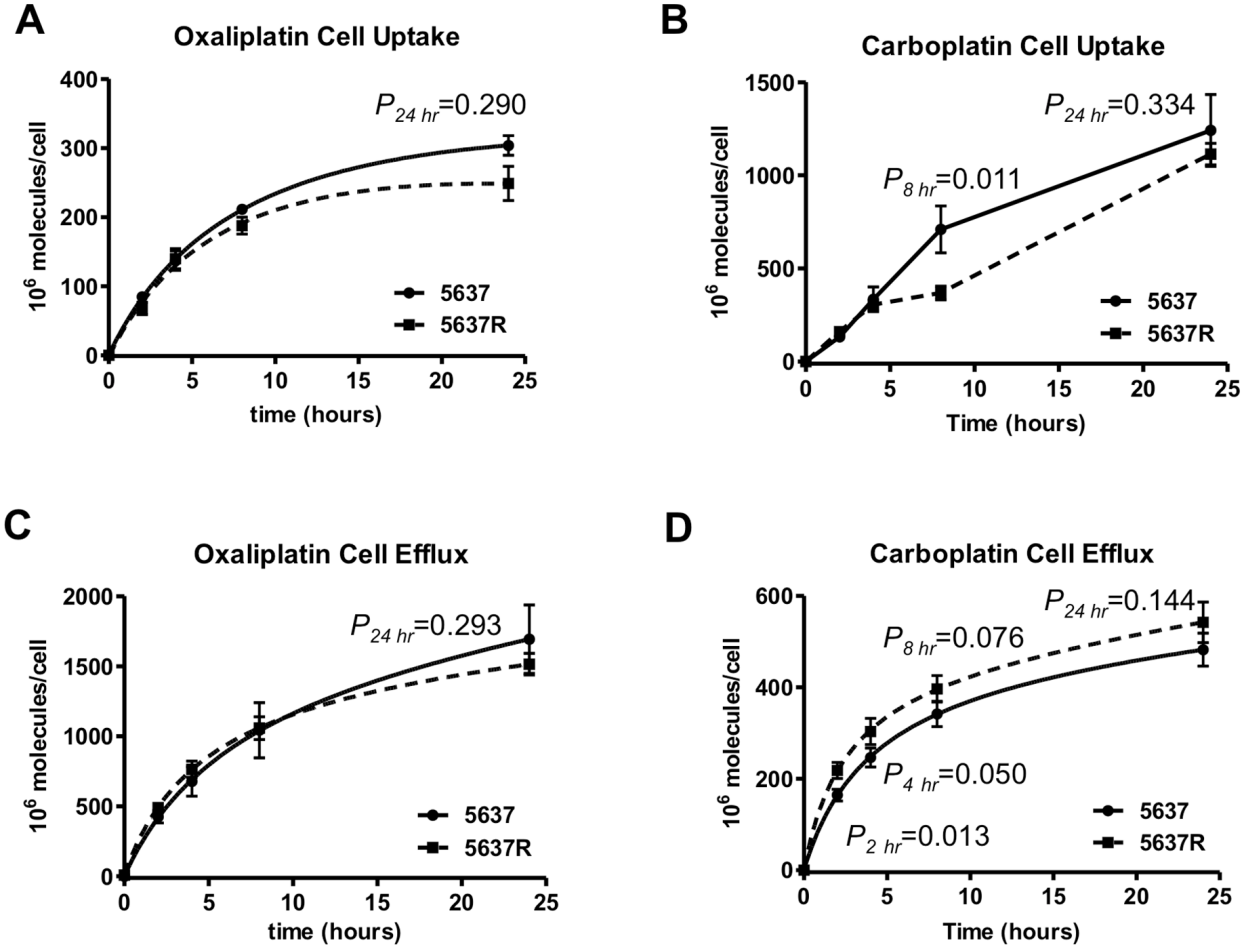
Drug uptake and efflux. (A-B) Comparison of cell uptake and efflux. A. cell uptake of oxaliplatin. 5637R cells had decreased cell uptake. B: 5637 and 5637R had similar cell efflux rates. (C-D) Oxaliplatin and carboplatin cellular efflux differences between the two cell lines were not statistically significant.

To determine drug uptake, cells were incubated with [^14^C]oxaliplatin or [^14^C]carboplatin, and sampled at various time points over 24 hours followed by isolation of cells and LSC analysis of intracellular drug accumulation. The 5637R line had a modest, but significantly lower peak intracellular oxaliplatin level at 24 hours (248.6 ± 24.7 X 10^6^ molecules per cell versus 303.7 ± 14.2 X 10^6^ molecules per cell for 5637 cells, p = 0.290) (Fig 2A). In contrast, both cell lines had similar levels of carboplatin uptake at 24 hours (1241 ± 192 X 10^6^ molecule per cell versus 1113 ± 58 X 10^6^ molecule per cell, p = 0.334), (Fig 2B).

To determine drug efflux, cells were exposed to [^14^C]oxaliplatin or [^14^C]carboplatin for 4 hours, washed with PBS and cultured in drug-free medium for 24 hours. The culture medium was sampled by LSC at different time points for determination of the rate of efflux. There was no significant difference in oxaliplatin or carboplatin efflux between the two cell lines (the efflux at 24 hours was 1514 ± 78 X 10^6^ molecules versus 1693 ± 244 X 10^6^ molecules per cell for oxaliplatin, p = 0.293 (Fig 2C); and 542.3 ± 44.5 X 10^6^ molecules versus 482.5 ± 35.9 X 10^6^ molecules per cell for carboplatin, p = 0.14) (Fig 2D).

### Intracellular inactivation

**Fig 3:**
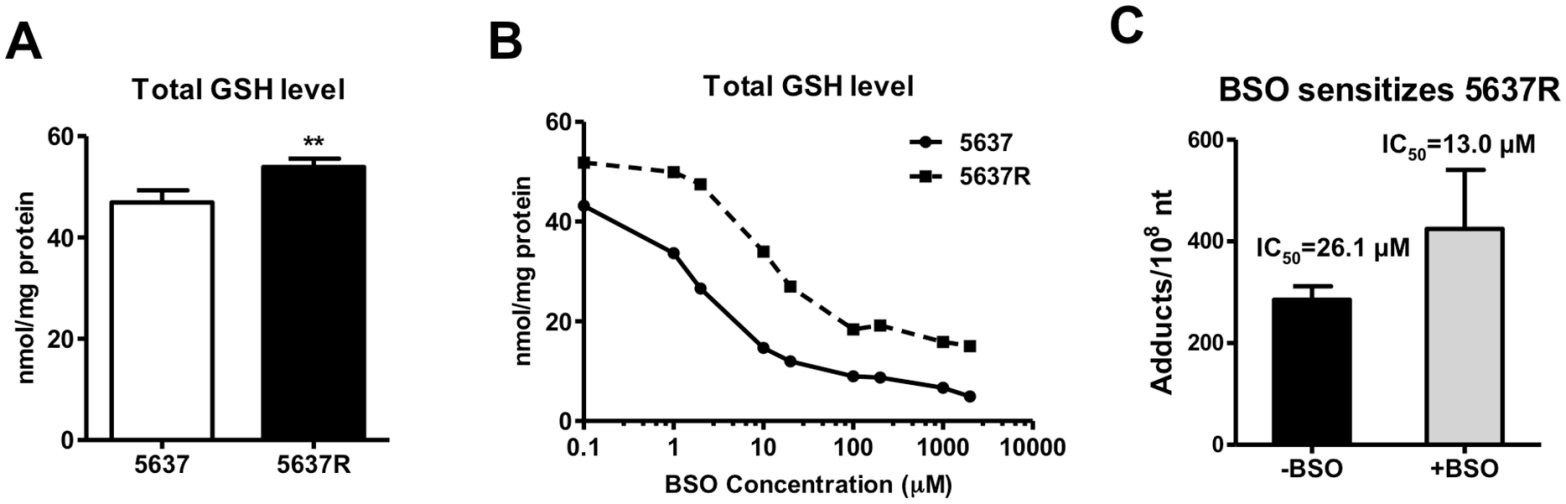
Drug inactivation by cellular glutathione. (A) Comparison of carboplatin-DNA adduct formation between 5637 and 5637R. (B) Comparison and correlation of IC_50_ values with carboplatin-adduct AUC, adduct levels four hours after dosing and DNA repair. (C) Comparison of cell uptake and efflux of carboplatin between 5637 and 5637R cells.

5637R cells had a significantly higher mean GSH concentration than 5637 cells (53.91 ± 0.83 nmol/mg protein versus 46.93 ± 1.20 nmol/mg protein. p = 0.003) (Fig 3A). To determine if the higher GSH concentration contributed to chemoresistance, both cell lines were cultured in the presence of buthionine sulphoximine (BSO), an inhibitor of gamma-glutamylcysteine synthetase, which is required for GSH biosynthesis [40]. BSO treatment decreased GSH in both cell lines in a dose-dependent manner (Fig 3B). Exposure of 5637R cells to 50 μM BSO followed by [^14^C]oxaliplatin exposure increased mean oxaliplatin-DNA adduct levels at 24 h from 285.4 ±15.3 adducts per 10^8^ nucleotides to 424.6 ± 67.7 adducts per 10^8^ nucleotide, but this was not statistically significant (p = 0.113). However, BSO treatment significantly decreased the oxaliplatin IC_50_ from 26.08 μM for 5637R cells to 12.95 μM for BSO treatment (p = 0.002, Fig 3C). In contrast, BSO treatment had little effect on the sensitivity of 5637 cells to oxaliplatin (IC_50_ of 2.45 μM untreated versus 2.36 μM with BSO exposure, data not shown. Unexpectedly, BSO exposure had no impact on the carboplatin IC_50_ values for either cell line (data not shown).

### Drug-DNA adduct formation and repair

**Fig 4:**
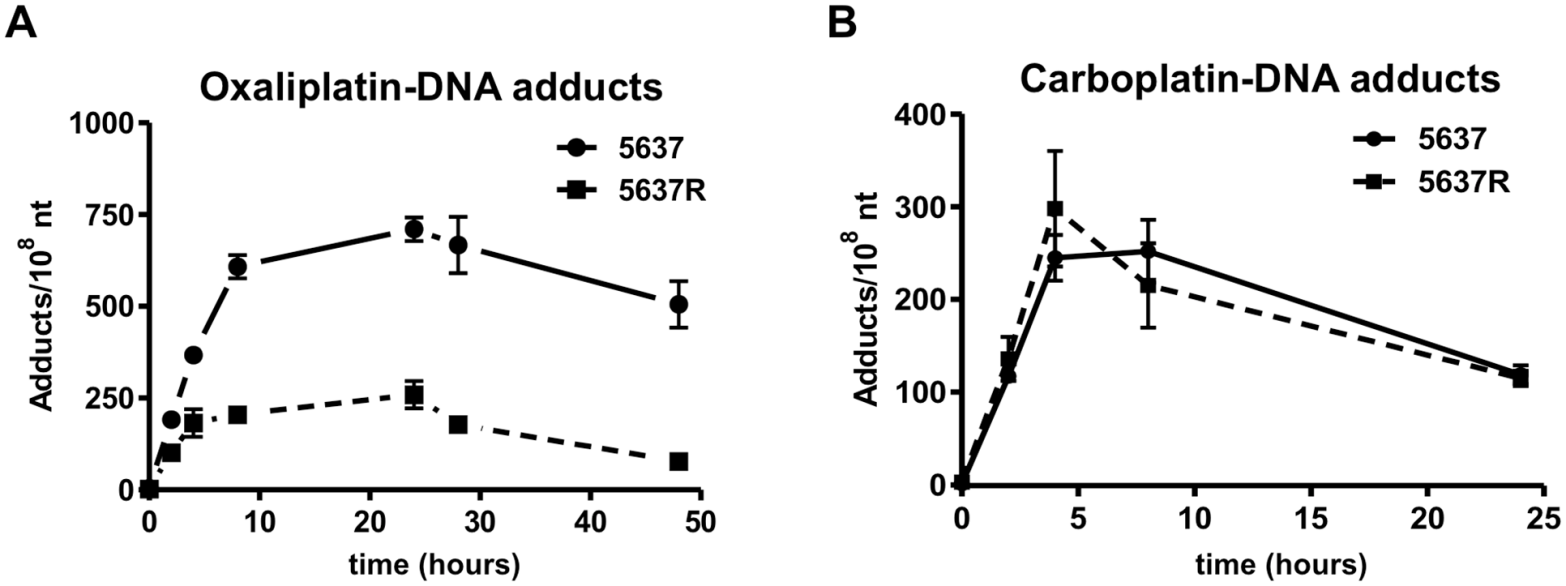
Oxaliplatin- and carboplatin-DNA adduct formation and repair. Comparison of oxaliplatin- and carboplatin-DNA adduct formation between 5637 and 5637R cells. The chemoresistant 5637R cells had higher oxaliplatin-DNA adduct levels at all time points compared to more treatment sensitive 5637 cells.

Cells were cultured with [^14^C]oxaliplatin at 10 μM (the approximate peak human oxaliplatin plasma concentration during chemotherapy) for 24 hours, followed by washing and culture for an additional 24 hours [33]. This protocol crudely mimics the *in vivo* exposure of oxaliplatin (exponential decrease blood concentration over approximately a day). Cells were harvested at various time points over 48 hours for DNA extraction and accelerator mass spectrometry (AMS) analysis using methods previously reported [41]. Briefly, AMS works by breaking down the molecules in a sample into atoms that are then identified and quantified in a small particle accelerator [42]. If the sample is labeled with a rare isotope such as ^14^C, the concentration of radiocarbon atoms in the particle beam can be used to calculate the concentration of drug in blood, tissue, cells and sub cellular components such as protein and DNA. AMS analysis typically requires the conversion of samples to graphite prior to analysis, which can be done using a high throughput parallel process. There was a time-dependent increase in oxaliplatin-DNA adduct levels during a 24-hour incubation, followed by a gradual decrease over the subsequent 24 hours owing to a combination of DNA repair and dilution of the signal by DNA synthesis. At all time points, the oxaliplatin-DNA adduct levels in 5637R cells were lower than the adduct levels in 5637 cells (Fig 4A). At 48 hours, 5637R cells had much lower DNA adducts than the parental 5637 cells (78 ± 4 versus 505 ± 63 adducts per 10^8^ nucleotide, p<0.0001, Table 2). The AUC of oxaliplatin-DNA adducts integrated over the 48 hours study time was significantly lower for 5637R cells (9,426 ± 2457 adducts-hr per 10^8^ nucleotides versus 27,720 ± 2,985 adducts-hr per 10^8^ nucleotides, p = 0.001, Table 2).

**Table 2:** Oxaliplatin-DNA adduct formation and repair.

Cell lines	48h	AUC_0-48h_	DNA repair
	(Adducts/10^8^ nt)	(Adducts/10^8^ nt⋅hour)	(Adducts/10^8^ nt/hour)
**5637**	505±63	27,720 ± 2,985	1.34 ± 0.30
**5637R**	78±4	9,426 ± 2,457	3.48 ± 0.15
***P***	*0*.*001*	*0*.*0012*	*0*.*0004*

For DNA repair studies, cells were exposed to [^14^C]oxaliplatin for 24h, washed and cultured further in oxaliplatin-free medium. The decrease of oxaliplatin-DNA adducts at several time points over the next 24 hours was used to calculate the drug-DNA adduct repair velocity. 5637R cells had a repair rate of 3.48 ± 0.15 adducts per 10^8^ nucleotides/hour and 1.34 ± 0.30 adducts per 10^8^ nucleotides-hour for 5637 cells (p = 0.0004, Table 2).

The formation and repair of carboplatin-DNA adducts was similarly determined, but with shorter drug exposures (4 hour exposure followed by washing and a twenty hour incubation in drug-free media) in order to mimic the faster *in vivo* plasma half-life compared to oxaliplatin. Carboplatin-DNA adduct levels and drug-DNA repair rates were not significantly different between the two cell lines (AUCs of 4,527 ± 895 versus 4,211 ± 1,678 monoadduct-hr/10^8^ nucleotides, p = 0.69, Table 2; and drug-DNA repair rates of 6.30 ± 3.10 versus 9.31 ± 6.74 adducts/10^8^ nucleotides/hour, p = 0.34 for 5637R and 5637 cells, respectively, (Fig 4B).

### RNAseq analysis of 5637 and 5637R cells

**Fig 5:**
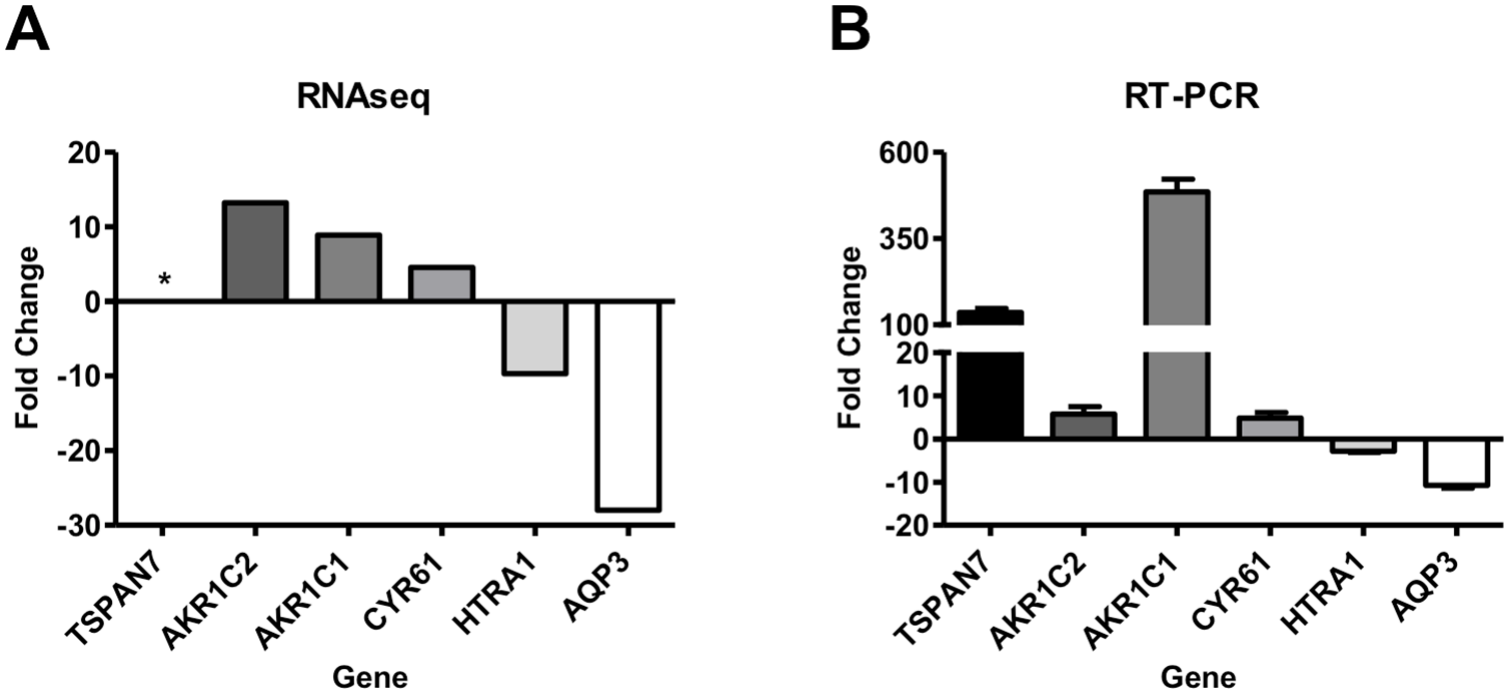
RNAseq and qRT-PCR show similar trends in gene expression levels for selected resistance-realated genes. Four genes (TSPAN7, AKR1C2, AKR1C1, and CYR61) have increased levels in 5637R cells and two genes (HTRA1 and AQP3) have decreased levels in the resistant cells. (A) Fold changes in chemoresistance gene levels relative to the 5637 parental cells as determined by RNA-seq. (B) Fold change in chemoresistance gene transcript levels relative to the 5637 parental cells as determined by qRT-PCR.

Total RNA was isolated from subconfluent cells that were cultured in the absence of chemotherapy drugs, and used for analysis by RNA-seq. From duplicate independent experiments, there were a total of 83 RNAs with statistically significant expression changes, of which 50 were associated with known genes, one known microRNA and 22 novel transcripts (p < 0.05, S1 Fig and S1 Table). The remaining 10 transcripts represent genes that did not provide measurable expression in all replicates, but may still be of relevance to drug resistance. Four genes of known relevance to chemoresistance (TSPAN7, AKR1C2, AKR1C1, and CYR61) were shown to be significantly upregulated in the resistant cell line 5637R and two genes (HTRA1 and AQP3) were downregulated compared to the parental cell line 5637 (Table 3). The RNA-seq results were further confirmed by qRT-PCR analysis of selected transcripts of RNA isolated from subconfluent cultures grown without drugs in duplicate (Fig 5).

**Table 3:** Chemoresistance-associated gene expression levels in 5637 and 5637R cells.

Gene Symbol	Description	5637 FPKM	5637R FPKM	Fold Change	P value	Q value
**Upregulated Genes**						
TSPAN7	tetraspanin 7	0	0.8041	—	0.00005	0.0130415
AKR1C2	aldo-keto reductase family 1, member C2	3.22761	42.6636	13.21832563	0.00005	0.0130415
AKR1C1	aldo-keto reductase family 1, member C1	5.67159	50.3706	8.881213205	0.00005	0.0130415
CYR61	cysteine-rich, angiogenic inducer, 61	3.92516	17.8771	4.554489499	0.0001	0.0216746
**Downregulated Genes**						
HTRA1	HtrA serine peptidase 1	23.9727	2.47473	-9.686996157	0.00005	0.0130415
AQP3	aquaporin 3	27.1631	0.97132	-27.96514022	0.0001	0.0216746

## Discussion

After ten months of cell culture under pressure from oxaliplatin exposure, the resulting resistant cell line retained a 5637 lineage as determined by STR analysis, which justified its designation as 5637R. Our results are comparable to other previous findings on parental and oxaliplatin resistant cancer cell lines cells [43–46]. It is puzzling and surprising that carboplatin exposure of 5637 cells under similar experimental conditions did not produce a carboplatin resistant cell line, even though 5637R cells are significantly resistant to carboplatin. This is an unexpected result considering the nearly universal clinical onset of resistance in advanced cancers upon treatment with platinum-based regimen. The difficulty of inducing carboplatin resistance in 5637 cells may be due to the known poor cross resistance between oxaliplatin and carboplatin or cisplatin [2]. Oxaliplatin may induce a different set of mutation spectra compared to carboplatin, which has been reported in an *Hprt* gene mutation assay in CHO cells for a comparison between cisplatin and oxaliplatin [47]. Regardless of how they were generated, the resulting pair of cell lines represents a useful model system for induced drug resistance, especially considering their relatively few phenotypic and genotypic differences.

The 5637R/5637 pair was evaluated by the MTT assay for resistance to oxaliplatin and several drugs that are used in the treatment of bladder cancer. Oxaliplatin, carboplatin and gemcitabine were significantly less cytotoxic to 5637R cells. Doxorubicin, methotrexate and vinblastine were equally toxic to both cell lines. This result is not surprising considering acquired resistance to one drug often selects for one or more mechanistic pathways that have multiple drugs as substrates. In the clinic, response to first line platinum-based therapy is 40–50% for muscle invasive bladder cancer, but once resistance ensues, subsequent treatments have just 10–25% response rates [48, 49]. Although much additional work is needed, the MTT data from our model system indicate that it is feasible to have substantial cytotoxic response to subsequent chemotherapy after the onset of platinum drug resistance if the correct treatment is selected.

Perhaps the most common drug resistance mechanism is modulation of intracellular drug accumulation via changes in the rate of uptake and efflux [42]. 5637R and 5637 cell lines were characterized for drug uptake and efflux by measuring radiocarbon content in cells over time (uptake) and accumulation of radiocarbon in media (efflux) after exposure of cells to either [^14^C]oxaliplatin or [^14^C]carboplatin. There was significantly lower drug uptake in 5637R cells for oxaliplatin, but not for carboplatin (248.6 ± 24.7 X 10^6^ oxaliplatin molecules per cell versus 303.7 ± 14.2 X 10^6^ molecules per cell for 5637 cells, p = 0.029) (Fig 2A). Efflux was not significantly different for both cell lines regardless of whether they were exposed to oxaliplatin or carboplatin. These data support a contributing role for decreased drug uptake as a factor in the oxaliplatin resistance of 5637R cells. However, these observations do not explain increased carboplatin and gemcitabine resistance.

Since platinum drugs act as electrophiles to chemically bind to DNA, they are subject to intracellular inactivation by cellular antioxidants such as GSH. 5637R cells have significantly higher GSH levels than 5637 cells, indicating increased intracellular inactivation as a possible resistance mechanism. Exposure of 5637R to BSO reduced GSH, increased oxaliplatin-DNA adduct frequency and increased oxaliplatin cytotoxicity. In contract, BSO treatment had little effect on the sensitivity of 5637 cells to oxaliplatin. We previously observed these phenomena for carboplatin treatment in a variety of cancer cell lines [50]. It is difficult to understand why the 5637R cell line would have such a drastic difference in response to BSO treatment compared to 5637 cells. There is almost certainly a basal level of GSH required to maintain the net reducing environment needed for normal cell function, and GSH concentrations in excess of this level may be disproportionately able to contribute to drug resistance for electrophilic compounds such as the platinum drugs.

The level of drug-DNA adducts is the primary pharmacodynamic endpoint of platinum-based chemotherapy, and can vary substantially amongst different cell lines and tumors depending on a wide variety of known and unknown factors [42]. Under identical experimental conditions, 5637R cells exposed to oxaliplatin had significantly lower peak and overall oxaliplatin-DNA adducts over 48 hours. The repair rate of oxaliplatin-DNA adducts was also faster in 5637R cells, indicating DNA repair as an additional resistance mechanism. Carboplatin-DNA adduct formation and repair was not significantly different between the two cell lines, indicating yet additional carboplatin resistance mechanisms beyond those leading up to and including drug-DNA adduct formation and repair.

ERCC1 (Excision Repair Cross-Complementation Group 1) is a protein mainly involved in nucleotide excision repair, and increased ERCC1 expression is clinically associated with resistance to platinum [51, 52]. We previously showed that inhibition of ERCC1 expression in lung cancer cells increased drug-DNA adduct formation and reduced repair of carboplatin-DNA monoadducts and partially reversed chemoresistance [50]. In this study, we saw no significant difference in transcripts related to ERCC1 in the RNAseq data, even though there was differential sensitivity to carboplatin. However, we did observe a significant difference in the rate of repair of oxaliplatin-DNA adducts, which could not be accounted for by differences in nucleotide excision repair capacity, at least not by mRNA expression. This example highlights the difficulty of finding generally applicable markers of platinum resistance.

In addition to functional analysis, we also performed RNA-seq followed by targeted qRT-PCR experiments with 5637 and 5637R cell lines cultured in the absence of drugs in order to simulate the patient between cycles of chemotherapy. Of the fifty transcripts that represent known genes, alterations in transcript levels were observed for six putative chemoresistance genes. Four genes, TSPAN7, AKR1C1, AKR1C2, and CYR61 have elevated expression levels in the resistant cell line compared to the parental cells. Tetraspanin 7 (TSPAN7) is a transmembrane protein involved in signal transduction. Increased levels of TSPAN7 in patients with acute lymphoblastic leukemia, chronic myeloid leukemia, or acute myeloid leukemia are associated with drug resistance [53]. AKR1C1 and AKR1C2 (aldo-ketose reductase family 1, members C1 and C2) or dihydrodiol dehydrogenase (DHH) are part of the progesterone metabolism pathway. Increased AKR1C1/2/3 levels are observed in cisplatin-resistant bladder cancer and colon cancer cell lines, leukemia cells continuously treated with daunorubicin, and in cisplatin-resistant ovarian cancers [54–57]. Transfection of the AKR1 genes into cell lines enhances chemoresistance while siRNA knockdown or inhibition of the genes resensitizes the cells to drug treatment [54–56]. A decreased level of ROS production was observed in the resistant cell lines treated with cisplatin. Since AKR1 is involved in cellular response to ROS, this result suggests that the resistance may be due to anti-oxidative effects [55, 56]. Additionally, the pro-inflammatory pathway was shown to increase AKR1C1/2 levels in non-small cell lung cancer cells leading to cisplatin and doxorubicin resistance [58]. CYR61 is an extracellular matrix-associated protein that mediates cell proliferation, angiogenesis, and adhesion. Overexpression of CYR61 in breast cancer cells caused resistance to paclitaxel and PI3K pathway inhibitors, suggesting activation of the pro-survival PI3K pathway as a mechanism for resistance [59]. Similarly, overexpression of CYR61 in pancreatic cancer cells led to reduced gemcitabine sensitivity that could be reversed by siRNA knockdown of CYR61 [60]. Though further investigations are needed, these studies support the possibility of using TSPAN7, AKR1C1/2, and CYR61 as biomarkers for resistance and that knockdown or inhibition of these genes may prevent or reduce platinum chemoresistance in bladder cancer.

Conversely, we found two genes, HTRA1 and AQP3, with decreased expression in the resistant cell line compared to the parental cells. HTRA1 is a member of the serine protease family and a potential tumor suppressor that is downregulated in several cancer types. Low levels of HTRA1 attenuate cisplatin and paclitaxel toxicity in ovarian cancer cells treated with HTRA1 siRNA and developed cisplatin-resistant non-small cell lung cancer cell lines and xenografts [61, 62]. Reexpression or overexpression of HTRA1 in the cell lines and xenografts improved drug sensitivity [61–63]. Similar findings were observed in ovarian and gastric cancer patients where high HTRA1 levels correspond to the best response to cisplatin-based therapies [61, 64]. Activation of the PI3K pathway is observed in low-HTRA1 NSCLC cells and inhibition of this pathway resensitizes cells to cisplatin treatment, suggesting one mechanism of resistance and potential treatment strategy [62]. Aquaporin 3, AQP3, is a small integral membrane protein involved in water transport across the plasma membrane. Similar to our findings, AQP3 levels were decreased in a cisplatin-resistant bladder cancer cell line compared to the parental cells [65]. Inhibition of AQP3 in bladder cancer cell lines or knockdown of AQP3 in breast and colon cancer cell lines decreases intracellular platinum concentration and attenuates the pro-apoptotic effects of nucleoside analog (5’ DFUR and gemcitabine) treatment, respectively [65, 66]. Low levels of HTRA1 and AQP3 are potential biomarkers for chemoresistance. Of course, increasing the levels of these proteins in tumors is a more difficult task than decreasing the levels of overexpressed or aberrantly activated genes. Understanding the mechanism of decreased expression could identify a potential means of reversing or reducing the chemoresistance, such as inhibition of the PI3K pathway in NSCLC cells with low HTRA1 expression. A few of the genes identified have been associated with alterations epithelial-mesenchymal transition (EMT) pathways, which have also been implicated in platinum chemoresistance in bladder cancer. These include NKX1-2, CHD8 and MFAP5, which may be potentially useful signatures of resistance [67–69].

The importance of non-coding RNAs, particularly long noncoding RNAs (lncRNAs), in cancer development has been increasingly acknowledged [70]. Among the 22 novel transcripts showing significant differential expression, only one 158 bp sequence can be classified as putative small regulatory RNAs. Most range in size between 300 bp-7 kb, several are 11–14 kb in length and a few are very large, from 38 kb up to 91 kb (see S2 Table). It is interesting that novel transcripts with non-zero fragments per kilobase of exon per million fragments mapped (FPKM) in both cell types are much longer than those with zero FPKM in one cell type. Additional experiments will be required to validate these transcripts and further explore their potential roles in bladder cancer development.

## Conclusions

Resistance to chemotherapy is a highly complicated process. The cytotoxic nature of non-targeted cytotoxic drugs results in the alteration of the expression of hundreds of genes [30]. In order to address the issue of genetic complexity in a model system, we undertook a functional and molecular analysis of two closely related cancer cell lines as a model system of drug resistance in bladder cancer. We performed a phenotypic and genotypic assessment of the major mechanistic pathways involved in the acquired chemoresistance of the 5637R cell line. The goal was to simplify mechanistic studies in a novel bladder cancer model system, but also to lay the foundation to someday guide the selection of therapeutic agents to overcome resistance specifically in the bladder cancer setting.

The phenotypic analysis indicated that drug accumulation, intracellular inactivation, drug-DNA damage and repair all contributed to oxaliplatin resistance in 5637R cells, whereas the RNAseq data revealed a contribution to post-DNA damage pathways related to many cancer-relevant processes such as apoptosis, growth signaling and others. Interestingly, there was not a clear link between the transcript expression levels and the observed differential repair of drug-DNA adducts. Perhaps subtle gene expression level differences or other regulatory mechanisms are important in regulating DNA repair as part of acquired drug resistance. Taken together, these seemingly disparate functional and molecular data sets form a foundation for additional novel investigations into chemoresistance. Most of the genes identified in the RNAseq analysis are novel for bladder cancer, which provides an opportunity for new testable hypotheses to tie these novel resistance genes to the now canonical phenotypic resistance pathways.

In conclusion, we have developed a nearly isogenic pair of cell lines that can be used to study chemoresistance in bladder cancer. Functional and mRNA expression analysis elucidated the major resistance pathways demonstrating feasibility for use of this model system as a tool to study the influence of specific gene expression or mutation differences to improve our understanding of drug resistance with the goal of ultimately guiding the design of new chemotherapy diagnostics and treatments. Future work will focus on functional assessment of the candidate transcripts identified in this report and on assessment of transcript expression changes in these cell lines upon platinum drug exposure.

## Supporting Information

S1 Fig Differentially expressed transcripts for 5637 and 5637R cells. (TIFF)

S1 Table Differentially expressed known transcripts. (PDF)

S2 Table Differentially expressed novel transcripts. (TIFF)
