# Supplementary figures and images for: Molecular Dissection of Induced Platinum Resistance through Functional and Gene Expression Analysis in a Cell Culture Model of Bladder Cancer
Source: PLoS One. 2016 Jan 22;11(1):e0146256. doi: 10.1371/journal.pone.0146256 (PMC4723083; doi:10.1371/journal.pone.0146256)

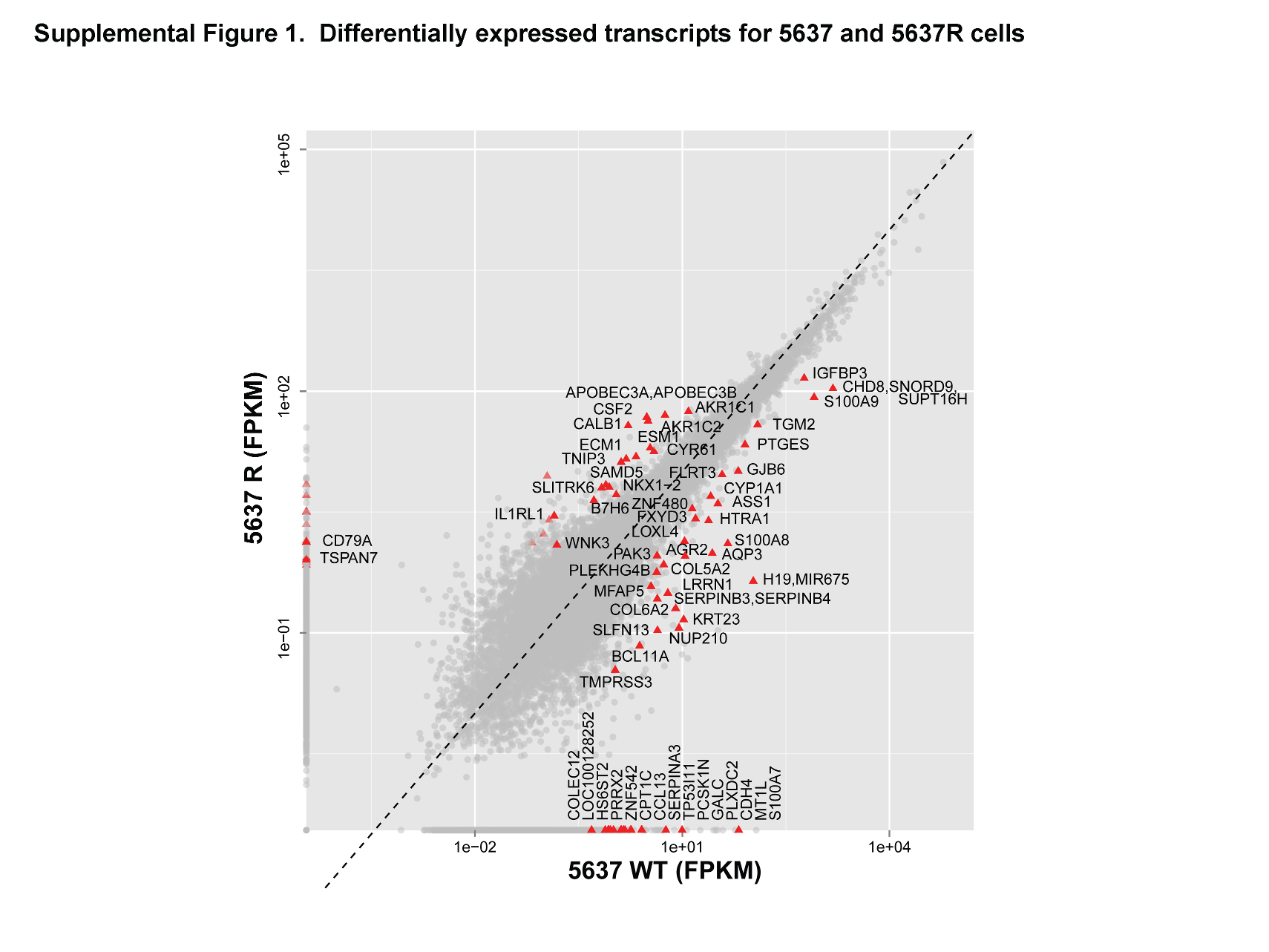

Supplement: S1 Fig — (TIFF) [file pone.0146256.s001.tiff]

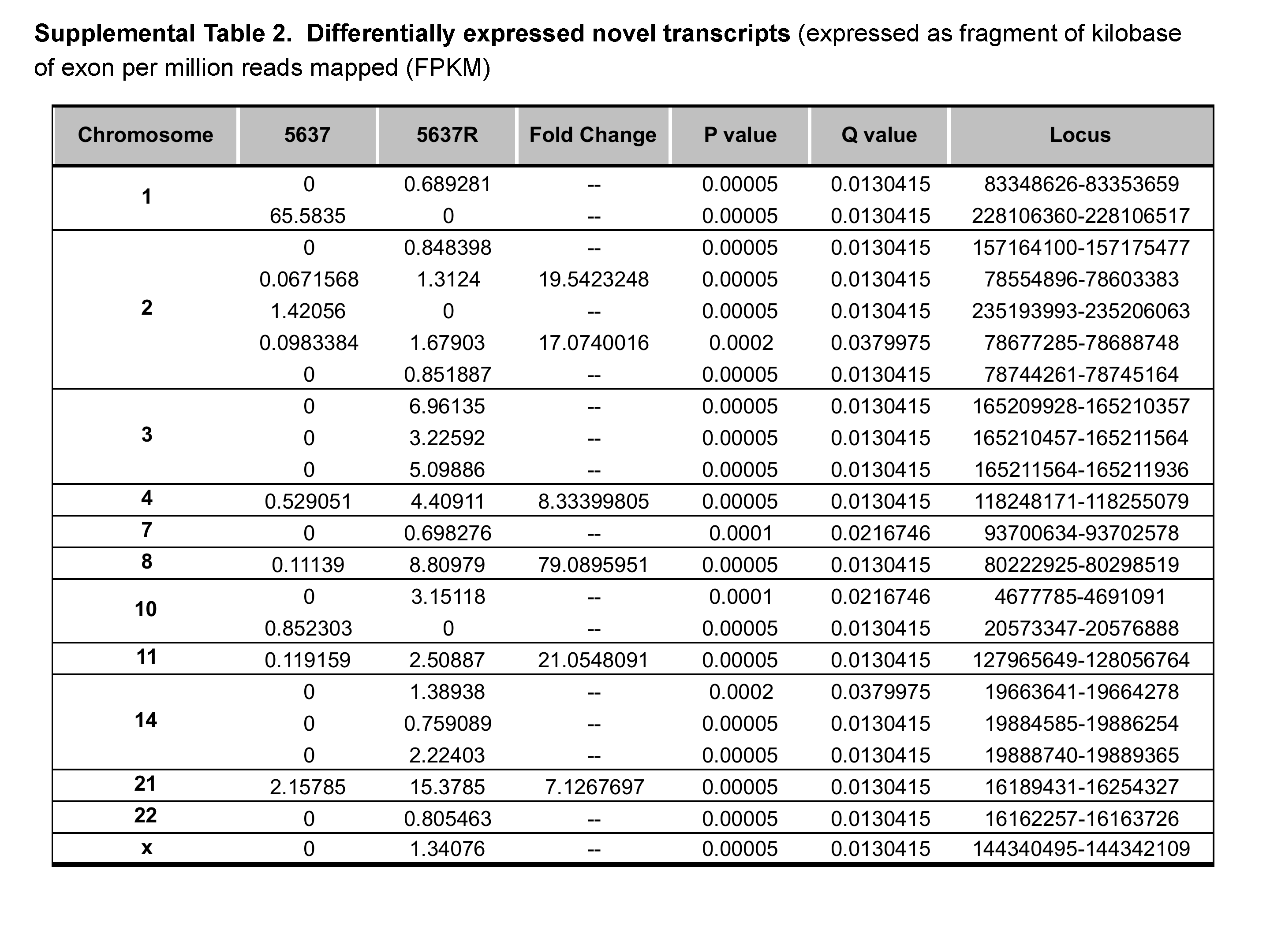

Supplement: S2 Table — (TIFF) [file pone.0146256.s003.tiff]
