# Supplementary material for: Molecular Dissection of Induced Platinum Resistance through Functional and Gene Expression Analysis in a Cell Culture Model of Bladder Cancer
Source: PLoS One. 2016 Jan 22;11(1):e0146256. doi: 10.1371/journal.pone.0146256 (PMC4723083; doi:10.1371/journal.pone.0146256)
Supplement: S1 Table — (PDF) [file pone.0146256.s002.pdf]

# Supplemental Table 1. Differentially expressed known transcripts

| gene name | 5637 | 5637R | fold change | fold change (log2) | test stat | p value | q value | comment |
|---|---|---|---|---|---|---|---|---|
| CALB1 | 1.66029 | 37.3762 | 22.51 | ↑ 4.49 | 4.72 | 0.00 | 0.01 | CALB1, Calbindin , Buffers cytosolic calcium. May stimulate a membrane Ca(2+)-ATPase and a 3',5'-cyclic nucleotide phosphodiesterase. Interacts with RANBP. The neurons in brains of patients with Huntington disease are calbindin-depleted. |
| IL1RL1 | 0.140789 | 2.83655 | 20.15 | ↑ 4.33 | 3.06 | 0.00 | 0.02 | Member of the interleukin 1 receptor family, can be induced by proinflammatory stimuli, and may be involved in the function of helper T cells. |
| CSF2 | 3.09079 | 47.7356 | 15.44 | ↑ 3.95 | 2.67 | 0.00 | 0.03 | Colony stimulating factor 2, Oncogenic Kras-induced GM-CSF production promotes the development of pancreatic neoplasia. |
| AKR1C2 | 3.22761 | 42.6636 | 13.22 | ↑ 3.72 | 3.72 | 0.00 | 0.01 | Selective reduction of AKR1C2 in prostate cancer and its role in DHT metabolism. |
| TNIP3 | 1.31294 | 13.1383 | 10.01 | ↑ 3.32 | 3.33 | 0.00 | 0.01 | TNFAIP3 interacting protein 3, possible role in immunity. |
| ECM1 | 1.55477 | 14.3977 | 9.26 | ↑ 3.21 | 3.23 | 0.00 | 0.02 | Encodes a soluble protein that is involved in endochondral bone formation, angiogenesis, and tumor biology. Overexpression of ECM1 contributes to migration and invasion in cholangiocarcinoma cell. |
| SLITRK6 | 0.684538 | 6.29545 | 9.20 | ↑ 3.20 | 3.10 | 0.00 | 0.01 | Controls neurite outgrowth. Overexpressed specifically in bladder, lung, breast and glioblastoma cancer. Agensys is developing an MMAE ADC for bladder cancer treatment with an anti-SLITRK6 antibody. |
| AKR1C1 | 5.67159 | 50.3706 | 8.88 | ↑ 3.15 | 3.87 | 0.00 | 0.01 | Reduces biologically active progesterone and 5α-pregnan-3α-ol-20-one into their corresponding 20α-hydroxysteroids among the isoforms, enzyme's over-expression in the cells of lung, ovary, uterine cervix, skin and colon carcinomas was reported to be associated with resistance against several anticancer agents. |
| SAMD5 | 0.790041 | 6.84632 | 8.67 | ↑ 3.12 | 3.45 | 0.00 | 0.01 | Sterile alpha motif domain containing 5, SAMD5 gene promoter binding sites: Olf-1 STAT5A NF-E2 p45 AREB6 POU2F1 POU2F1a Pax-4a NF-kappaB1. |
| WNK3 | 0.154106 | 1.23671 | 8.03 | ↑ 3.00 | 2.77 | 0.00 | 0.01 | Belonging to the 'with no lysine' family of serine-threonine protein kinases, plays a role in the increase of cell survival in a caspase 3 dependent pathway. |
| NKX1-2 | 0.88344 | 6.42323 | 7.27 | ↑ 2.86 | 2.83 | 0.00 | 0.03 | A critical component of the gene regulatory network that operates downstream of Wnt/b-catenin signaling to regulate the formation of mesendoderm. |
| ESM1 | 3.45777 | 19.73 | 5.71 | ↑ 2.51 | 2.99 | 0.00 | 0.01 | ESM-1 is significantly overexpressed in colorectal cancer (CRC) patients, and can be used as a potential biomarker and a therapeutic target for CRC, ESM-1 overexpression in HCT-116 cells enhanced cell proliferation through the Akt-dependent activation of NF-kappa-B pathway. |

## Supplemental Table 1. Differentially expressed known transcripts (continued).

| gene name | 5637 | 5637R | fold change | fold change (log2) | test stat | p value | q value | comment |
|---|---|---|---|---|---|---|---|---|
| B7H6 | 1.11473 | 5.18194 | 4.65 | ↑ 2.22 | 2.86 | 0.00 | 0.01 | B7-H6; identification as a tumor cell surface molecule that binds NKp30, a receptor which triggers antitumor NK cell cytotoxicity and cytokine secretion. |
| APOBEC3A,APOBEC3B | 12.2366 | 56.0271 | 4.58 | ↑ 2.19 | 2.58 | 0.00 | 0.03 | Cytidine deaminase associated with aquired immunity, but also breast cancer mutation showers called kataegis. |
| CYR61 | 3.92516 | 17.8771 | 4.55 | ↑ 2.19 | 2.51 | 0.00 | 0.02 | Secreted protein interacts with several integrins and with heparan sulfate proteoglycans, also plays a role in cell proliferation, differentiation, angiogenesis, apoptosis, and extracellular matrix formation. |
| TGM2 | 122.918 | 38.3556 | 0.31 | ↓ -1.68 | -2.43 | 0.00 | 0.02 | Transglutaminases are enzymes that catalyze the crosslinking of proteins by epsilon-gamma glutamyl lysine isopeptide bonds, induced by retinoic acid, and appears to be involved in apoptosis. |
| PTGES | 80.9826 | 21.6988 | 0.27 | ↓ -1.90 | -3.41 | 0.00 | 0.01 | Prostaglandin E synthase, a tight cooperation between the EGF/EGFR and mPGES-1 leads to a significant tumorigenic gain in epithelial cells. |
| ZNF480 | 13.9051 | 3.47044 | 0.25 | ↓ -2.00 | -3.02 | 0.00 | 0.01 | Zinc finger protein 480, regulatory transcription factor binding sites in the ZNF480 promotor: STAT1 AP-1 ATF-2 STAT1beta STAT1alpha AREB6 Chx10 c-Jun ATF6 Ik-1. |
| IGFBP3 | 582.548 | 145.356 | 0.25 | ↓ -2.00 | -2.55 | 0.00 | 0.04 | Insulin-like growth factor binding protein (IGFBP)-3 is a pro-apoptotic and anti-angiogenic protein in prostate cancer (CaP). It is a metastasis supressing gene in prostate cancer. |
| FLRT3 | 37.6701 | 9.21929 | 0.24 | ↓ -2.03 | -2.88 | 0.00 | 0.02 | Fibronectin leucine rich transmembrane protein 3, FLRTs may function in cell adhesion and/or receptor signalling. |
| PAK3 | 4.3532 | 0.904633 | 0.21 | ↓ -2.27 | -2.64 | 0.00 | 0.02 | A serine-threonine kinase, role of p21-activated kinase 3 (PAK3) in activating protein 1 (AP-1) induced oncogenesis, plays a role in cell proliferation, differentiation and apoptosis, regulated by AP-1. |
| CYP1A1 | 25.7451 | 4.95149 | 0.19 | ↓ -2.38 | -3.30 | 0.00 | 0.02 | Cytochrome p450 detox enzyme that also mediates interaction of AhR with various biological pathways, including cell cycle control, apoptosis, mitogen-activated protein kinases, estrogen receptor, glucocorticoid receptor and hypoxia signaling. |
| FXYD3 | 15.5322 | 2.6126 | 0.17 | ↓ -2.57 | -2.74 | 0.00 | 0.04 | FXYD domain containing ion transport regulator 3, This gene encodes a cell membrane protein that may regulate the function of ion-pumps and ion-channels. This gene may also play a role in tumor progression. |
| GJB6 | 64.5047 | 10.1797 | 0.16 | ↓ -2.66 | -3.58 | 0.00 | 0.01 | Gap junction protein, beta 6, 30kDa, also known as Connexin 30. Gap junctions made with connexin 30 transport potassium ions and certain small molecules, associated with deafness. Possibly a pathway for drug import/export. |

## Supplemental Table 1. Differentially expressed known transcripts (continued).

| gene name | 5637 | 5637R | fold change | fold change (log2) | test stat | p value | q value | comment |
|---|---|---|---|---|---|---|---|---|
| PLEKHG4B | 4.30022 | 0.565512 | 0.13 | -2.93 | -3.67 | 0.00 | 0.01 | Pleckstrin homology domain containing, family G (with RhoGef domain) member 4B, Rho guanyl-nucleotide exchange factor activity, phospholipid binding. |
| COL5A2 | 5.44264 | 0.700975 | 0.13 | -2.96 | -3.19 | 0.00 | 0.01 | Collagen, type V, alpha 2, This gene encodes an alpha chain for one of the low abundance fibrillar collagens. |
| LOXL4 | 10.7479 | 1.37071 | 0.13 | -2.97 | -3.19 | 0.00 | 0.01 | Lysyl oxidase-like 4, Alternatively spliced lysyl oxidase-like 4 isoforms have a pro-metastatic role in cancer. May function as a negative feedback regulator of TGF-beta1 in cell invasion by inhibiting the metabolism of extracellular matrix (ECM) components. |
| ASS1 | 32.6738 | 4.01297 | 0.12 | -3.03 | -3.95 | 0.00 | 0.01 | Argininosuccinate synthase 1, catalyzes the penultimate step of the arginine biosynthetic pathway, In patients with osteosarcoma, reduced expression of ASS is not only a novel predictive biomarker for the development of metastasis, but also a potential target for pharmacologic intervention. |
| LRRN1 | 3.55689 | 0.377705 | 0.11 | -3.24 | -2.74 | 0.00 | 0.05 | Leucine rich repeat neuronal 1, N-MYC promotes cell proliferation through a direct transactivation of neuronal leucine-rich repeat protein-1 (NLRR1) gene in neuroblastoma |
| S100A9 | 810.919 | 84.1582 | 0.10 | -3.27 | -3.95 | 0.00 | 0.01 | S100 calcium binding protein A9, involved in the regulation of a number of cellular processes such as cell cycle progression and differentiation, associated with cystic fibrosis, S100A9 promotes the proliferation and invasion of HepG2 hepatocellular carcinoma cells via the activation of the MAPK signaling pathway. Knocking down S100A9 enhanced tumor cell invasion. Ligand for EMMPRIN, which promotes melanomametastasis. Potential biomarker for prediction of lymph node metastasis in gastric adenocarcinoma. |
| HTRA1 | 23.9727 | 2.47473 | 0.10 | -3.28 | -4.53 | 0.00 | 0.01 | HtrA serine peptidase 1, This gene encodes a member of the trypsin family of serine proteases. It has also been suggested to be a regulator of cell growth. HtrA1 may function as a tumor suppressor by controlling the epithelial-to-mesenchymal transition, and may function in chemotherapeutic responsiveness by mediating DNA damage response pathways. |
| AGR2 | 10.9547 | 0.895274 | 0.08 | -3.61 | -2.99 | 0.00 | 0.04 | Anterior gradient 2 homolog, In vitro, knockdown of CD147 or AGR2 decreased cellular proliferation, migration and invasion. In vivo, knockdown of CD147 or AGR2 expression decreased primary tumor growth as well as regional and distant metastasis. |
| CHD8,SNORD9,SUPT16H | 1521.31 | 107.947 | 0.07 | -3.82 | -6.51 | 0.00 | 0.01 | Chromodomain helicase DNA binding protein 8, a transcription repressor, binds beta-catenin and negatively regulates Wnt signaling pathway, small nucleolar RNA, C/D box 9, suppressor of Ty 16 homolog. |

# Supplemental Table 1.  Differentially expressed known transcripts  (continued).

| gene name | 5637 | 5637R | fold change | fold change (log2) | test stat | p value | q value | comment |
|---|---|---|---|---|---|---|---|---|
| SERPINB3,SERPINB4 | 4.38891 | 0.2639 | 0.06 | ↓ -4.06 | -3.08 | 0.00 | 0.01 | Serpin peptidase inhibitor, clade B (ovalbumin), member 3, SERPINB3 in the chicken model of ovarian cancer: a prognostic factor for platinum resistance and survival in patients with epithelial ovarian cancer and in patients. several microRNAs, specifically miR-101, miR-1668 and miR-1681 were discovered to influence SERPINB3 expression.  Strong expression of SERPINB3 protein was a prognostic factor for platinum resistance, and for poor progression-free survival. over-expressed in human hepatocellular carcinoma and in regenerating liver in mice has been shown to induce apoptosis resistance, epithelial-to-mesenchymal transition and increasing cellular invasion. |
| MFAP5 | 6.23769 | 0.311416 | 0.05 | ↓ -4.32 | -3.92 | 0.00 | 0.01 | Microfibrillar associated protein 5, associated with poor prognosis in advanced ovarian cancer, induce Notch1 extracellular domain dissociation and receptor activation, promotes angiogenesis. |
| AQP3 | 27.1631 | 0.97132 | 0.04 | ↓ -4.81 | -4.71 | 0.00 | 0.02 | Aquaporin 3 (Gill blood group), in addition to its water channel function, aquaporin 3 has been found to facilitate the transport of nonionic small solutes such as urea and glycerol, Loss of aquaporin 3 protein expression constitutes an independent prognostic factor for progression-free survival: an immunohistochemical study on stage pT1 urothelial bladder cancer.  Aquaporin 3 (AQP3) participates in the cytotoxic response to nucleoside-derived drugs, including gemcitabine. |
| TMPRSS3 | 1.07891 | 0.034306 | 0.03 | ↓ -4.97 | -1.28 | 0.00 | 0.04 | Transmembrane protease, serine 3, This gene is expressed in fetal cochlea and many other tissues, and is thought to be involved in the development and maintenance of the inner ear or the contents of the perilymph and endolymph.This gene was also identified as a tumor-associated gene that is overexpressed in ovarian tumors. |
| BCL11A | 2.43331 | 0.068694 | 0.03 | ↓ -5.15 | -2.75 | 0.00 | 0.02 | B-cell CLL/lymphoma 11A (zinc finger protein), also known as EVI9; CTIP1; ZNF856; HBFQTL5; BCL11A-L; BCL11A-S; BCL11a-M; BCL11A-XL, The corresponding mouse gene is a common site of retroviral integration in myeloid leukemia, and may function as a leukemia disease gene, in part, through its interaction with BCL6.  Directly activates RAG gene expression and V(D)J recombination. |
| S100A8 | 45.544 | 1.28344 | 0.03 | ↓ -5.15 | -4.09 | 0.00 | 0.01 | S100 calcium binding protein A8, a member of the S100 family of proteins containing 2 EF-hand calcium-binding motifs. S100 proteins are localized in the cytoplasm and/or nucleus of a wide range of cells, and involved in the regulation of a number of cellular processes such as cell cycle progression and differentiation.  S100A8/A9, produced by myeloid-derived suppressor cells, plays a role in myeloid-derived suppressor cells activation in gastric cancer patients, identifying S100A8/A9 as a potential target for gastric cancer treatment. |

## Supplemental Table 1. Differentially expressed known transcripts (continued).

| gene name | 5637 | 5637R | fold change | fold change (log2) | test stat | p value | q value | comment |
|---|---|---|---|---|---|---|---|---|
| COL6A2 | 8.03175 | 0.200142 | 0.02 | -5.33 | -4.89 | 0.00 | 0.01 | Collagen, type VI, alpha 2, This gene encodes one of the three alpha chains of type VI collagen, These domains have been shown to bind extracellular matrix proteins, an interaction that explains the importance of this collagen in organizing matrix components. |
| SLFN13 | 4.41597 | 0.10677 | 0.02 | -5.37 | -4.21 | 0.00 | 0.01 | Schlafen family member 13, a protein encoding sequence. |
| KRT23 | 10.453 | 0.145772 | 0.01 | -6.16 | -5.24 | 0.00 | 0.05 | Keratin 23 (histone deacetylase inducible), possibly contributes to SMAD4-mediated tumor suppression. |
| NUP210 | 8.93209 | 0.11483 | 0.01 | -6.28 | -4.94 | 0.00 | 0.01 | Nucleoporin 210kDa, Nucleoporins are the main components of the nuclear pore complex. |
| H19,MIR675 | 106.654 | 0.437982 | 0.00 | -7.93 | -2.56 | 0.00 | 0.01 | H19: imprinted maternally expressed transcript (non-protein coding), and functions as a tumor suppressor, The gene is located in an imprinted region of chromosome 11 near the insulin-like growth factor 2 (IGF2) gene. Upregulated H19 contributes to bladder cancer cell proliferation by regulating ID2 expression. Long non-coding RNA H19 increases bladder cancer metastasis by associating with EZH2 and inhibiting E-cadherin expression. Related to mir-675 via regulation. |
